# Health seeking behavior and associated factors among individuals with cough in Yiwu, China: a population-based study

**DOI:** 10.1186/s12889-021-11250-5

**Published:** 2021-06-16

**Authors:** Xiaoyan Sun, Shuying Luo, Lingqiao Lou, Hang Cheng, Zhen Ye, Jianwei Jia, Yina Wei, Jingbo Tao, Hanqing He

**Affiliations:** 1 Yiwu Center for Disease Control and Prevention, Yiwu, 322000 P.R. China; 2 Zhejiang Provincial Center for Disease Control and Prevention, Hangzhou, 310051 P.R. China

**Keywords:** Cough, Health seeking decision, Choice of medical facility

## Abstract

**Background:**

Previous studies have shown that a certain proportion of the population did not seek medical treatment after coughing, and understanding the potential reasons is crucial for disease prevention and control.

**Method:**

A population-based study was conducted with the probability proportional to population size sampling in Yiwu, Zhejiang, China. A total of 5855 individuals aged ≥15 years lived in Yiwu for more than 6 months were included. All participants completed a laptop-based questionnaire to collect detailed information by a face-to-face interview. Characteristics of individuals were described by categories of health seeking behavior using frequency and percentage. Univariate and multivariate logistic regression analyses were performed to estimate the associations of social-demographic and cough characteristics with health seeking behavior.

**Results:**

19.3% (1129/5855) of participants had a cough in the past month, 40% (452/1129) had sought medical treatment. Of these, 26.5% (120/452) chose hospitals at county level or above. Individuals aged ≥65 years old (OR = 2.25, 95% CI: 1.23, 4.12), female (OR = 1.57, 95% CI: 1.21, 2.06), living in rural areas (OR = 1.30, 95% CI: 1.003, 1.69), persistent cough for 3–8 weeks (OR = 2.91, 95% CI: 1.72, 4.92) and with more accompanying symptoms (P _trend_ < 0.001) were more likely to seek medical treatment, but those coughed for > 8 weeks were not (*p* > 0.5). Female (OR = 0.33, 95% CI: 0.21, 0.54) and people living in rural areas (OR = 0.57, 95% CI: 0.36, 0.92) were less likely to choose hospitals at county level or above while the higher educated were more likely to (OR = 3.29, 95% CI: 1.35, 8.02). Those who coughed for more than 2 weeks were more likely to choose hospitals at or above the county level. But the number of accompanying symptoms does not show any significant relationship with the choice of medical facility.

**Conclusion:**

The present study found that age, sex, living areas and features of cough were associated with health seeking behavior. It is worth noting that those who coughed for too long (e.g. > 8 weeks) were less likely to seek medical treatment. Targeted measures should be developed based on the key factors found in this study to guide persons to seek medical treatment more scientifically.

**Supplementary Information:**

The online version contains supplementary material available at 10.1186/s12889-021-11250-5.

## Background

Cough is an essential defensive mechanism. It is not only a common symptom of respiratory diseases but also can reflect significant diseases [1, 2]. More importantly, cough is an important factor in the spread of respiratory infectious diseases [3, 4]. According to the International Standards for Tuberculosis Care, all persons with otherwise unexplained productive cough lasting 2–3 weeks or more should be evaluated for tuberculosis [5, 6]. Recent studies also found that pertussis characterized by persistent cough is more prevalent in some areas, with a prevalence of 10.9–24.5% [7–9]. In China, the prevalence of cough fluctuates between 9 and 64%, highlight the level of significance and the corresponding burden of disease cannot be underestimated [10]. Thus scientific health seeking behavior of patients with cough is of great importance, which is conducive to the early diagnosis and treatment of diseases, especially contribute to the promptly prevention and control of respiratory infectious diseases.

However, previous studies found that a high proportion of patients who did not seek medical treatment after cough, and socio-demographic charactersistics were associated with health-seeking decision [11–13]. However, most of them were conducted abroad and found that evidence describing the potential risk factors are heterogeneous. A risk factor in one setting may promote the health-seeking decision but not in other settings. For example, a Ethiopia study found that higher income group were more likely to seek medical service [14], while studies in South African [12] and Zambia [15] showed an opposite association for the same risk factor, and study in India [16] found there was no correlation between income and health-seeking decision. The same situation occurs in other risk factors, such as age, sex, occupation and smoking status [13, 15, 17–19]. Besides, some factors were studied extensively in many studies (namely age, sex, education, occupation and income) while others may only appear in very few studies (namely, features of cough) [13, 20]. What’more, there is still no study has focused on the association between the duration of a cough and health seeking behavior.

Besides, studies on the choice of medical facilities for individuals with cough is very limited. In China, only very few studies have conducted at health facility level and focused on the individuals with presumptive tuberculosis [21–23].

Here, a community survey provided us with a unique opportunity to look at the health seeking behavior of individuals with cough. The aim of the present study was to describe the health seeking behaviors (including health-seeking decision and choice of medical facility) of individuals with cough, and to examine the associations of social-demographic characteristics and features of cough with health seeking behavior.

## Methods

### Study design and population

A cross-sectional survey was conducted in Yiwu—a county-level city of about 2,000,000 people—situated in the central Zhejiang Province of China. All individuals aged ≥15 years and were living in Yiwu for more than 6 months during the months of October to December 2019 were considered as the source population.

Fifty clusters were selected from 14 town/streets in Yiwu with the probability proportional to population size sampling method, followed by a quota sampling method based on age and sex to select 110 individuals from each cluster. Then well-trained investigators went to each cluster to interview individuals who met the above criteria through simple random sampling.

All individuals completed a laptop-based questionnaire to collect detailed information by a face-to-face interview after signing the informed consent. Any impermanent residents from the sampled village (residential community) who lived in Yiwu for less than 6 months were excluded. If there were two or more eligible people from one family, only one was randomly selected to participate in the survey. Individuals who have had a cough in the past month were included in the present study.

### Study questionnaire

We used a 12-item questionnaire to ask the cough status and health seeking behavior of the participants. The questionnaire was further revised after discussion and revision by the project team experts and on-site pre-investigation before it was officially used. Besides, logical verification was carried out in the design to avoid filling errors and missing information. The questionnaire consists of two parts. The first part included the basic information of participants, including age, sex, ethnic, occupation, education, family size, income, smoking status and history of chronic disease. The second part included the participants’ cough status in the past month, whether they went to a doctor because of the most recent cough, and which medical institution has been chosen. See additional files for details.

### Measurement

#### Assessment of cough and health seeking behavior

Cough was assessed by asking participants “Have you had a cough during the past month”. If the participants answered “Yes”, they were further asked the cough duration and whether they had accompanying symptoms, such as fever, expectoration, sore throat, runny nose, dyspnea, headache, fatigue, lethargy, earache, muscle or joint pain, abdominal pain, shortness of breath, chest pain, irritability and others. We categorized the duration of cough period into ≤2 weeks, 3–8 weeks and > 8 weeks, and defined ≤2 weeks as the reference group. And numbers of accompanying symptoms into 3 groups, namely 0 (reference), 1, 2 and above.

In the present study, we defined health seeking behavior as health-seeking decision and choice of medical facility. Participants were asked “Did you go to a medical facility for treatment duringyour last cough (not limited to the past month)?” to acquire their health-seeking decision. If the participants answered “Yes”, they were further asked “which medical facility did you choose”. Options include clinics, 14 township hospitals or community health service centers, municipal hospitals, and others (open answer). We categorized the choice of medical facility into hospitals at county level or above and community health service center or below.

### Other covariates

Other covariates including age (15–24, 25–64, ≥65 years old), sex, living areas (rural or urban), level of education (primary school or below, middle/high school, college or above), occupation (student, unemployment, business and services, professionals, farmers and workers), household income (< 100,000, 100,000–199,999 and ≥ 200,000 yuan per year), children under 5 years old (yes or no), and current smoking status (yes or no). Besides, they were further asked if they had a history of chronic diseases (yes or no) by question “whether you have the following chronic diseases: hypertension, cardiovascular and cerebrovascular diseases, diabetes, chronic diseases of ear, nose and throat, chronic respiratory diseases, digestive diseases, and others”.

### Statistical analysis

Socio-demographic characteristics of participants were described using frequency and percentage. Both univariate and multivariate logistic regression analyses were performed to estimate the associations of social-demographic characteristics and features of cough with health-seeking decision or choice of health facilities. All variables in the bivariate analyses were included in the multivariate analysis utilsing binary logistic regression analysis with enter process. All statistical analyses were conducted using SPSS 19.0 statistical software and all *p*-values refer to two-tailed tests. Statistical significance was set at *P* < 0.05.

## Results

### Characteristics of study population

A total of 5860 respondents participated in the interviews, of which 5 were excluded because they did not complete the survey.

Of 5855 eligible individuals interviewed, 69.6% of individuals aged 25–64 years old, 10.8% were ≥ 65 years old, 50.7% were females and 40.0% living in rural areas. Around half had attended middle school, 22.7% graduated from college and above. 1129 (19.3%) reported coughing in the past month. Detailed characteristics of individuals are shown in Table 1.

**Table 1:** Socio-demographic characteristics of the total population and individuals with cough in the past month

Characteristics	Total population		Individuals with cough	
	N	%	N	%
**Total**	5855	100	1129	19.3
**Age (years)**				
15-24y	1146	19.6	245	21.4
25-64y	4078	69.6	684	16.8
≥ 65y	631	10.8	200	31.7
**Residence**				
Urban	3512	60.0	671	19.1
Rural	2343	40.0	458	19.5
**Sex**				
Male	2887	49.3	576	20
Female	2968	50.7	553	18.6
**Occupation**				
Student	335	5.7	104	31
Unemployed	1461	25.0	337	23.1
Business/service	2104	35.9	314	14.9
Professional	1247	21.3	232	18.6
Farmers and workers	708	12.1	142	20.1
**Education**				
Primary school and below	1267	21.6	309	24.4
Middle school	3261	55.7	531	16.3
College and above	1327	22.7	289	21.8
**Child under 5 years old**				
No	3450	58.9	708	20.5
Yes	2405	41.1	421	17.5
**Income (yuan)**^a^				
< 100,000	3372	57.6	667	19.8
100,000-199,999	1772	30.3	331	18.7
≥ 200,000	710	12.1	130	18.3
**Smoking status**^a^				
No	3249	55.5	592	18.2
Yes	2605	44.5	537	20.6
**Chronic disease**				
No	4695	80.2	783	16.7
Yes	1160	19.8	346	29.8

^a^Missing data for one individual

### Factors associated with health-seeking decision

Of the 1129 participants who reported coughing in the past month, 40% (452) had been to medical facilities. The associations of socio-demographic characteristics and features of cough with health-seeking decision are presented in Table 2. Participants who aged ≥65 years old, female and living in rural areas were associated 2.26 (95% CI: 1.24, 4.14), 1.57 (95% CI: 1.21, 2.07) and 1.31 (95% CI: 1.01, 1.70) times greater likelihood of seeking medical treatment respectively. Compared with individuals coughed for ≤2 weeks, those coughed for 3–8 weeks was 2.81 (95% CI: 1.68, 4.73) times more likely to seek medical treatment. However, there was no significant difference between those coughed for more than 8 weeks and less than 2 weeks. A significant positive trend (*P* < 0.001) was observed between the number of accompanying symptoms and health-seeking decision. Compared with participants who reported no accompanying symptoms with cough, those reported symptoms were more likely to seek medical treatment, particularly for those with two symptoms and above (OR = 3.87, 95% CI: 2.74, 5.47).

**Table 2:** Univariate and multivariate logistic regression analysis for health-seeking decision after coughing (*N* = 1129)

Characteristics	Seeking treatment N (%)	Univariable		Multivariable	
		Crude OR	***P*** value	Adjusted OR	***P*** value
**Age**					
15-24y	86 (35.1)	Ref		Ref	
25-64y	267 (39.0)	1.18 (0.87,1.16)	0.277	1.44 (0.95,2.19)	0.089
≥65y	99 (49.5)	1.81 (1.24,2.65)	0.002	2.26 (1.24,4.14)	0.008
**Residence**					
Urban	247 (36.8)	Ref		Ref	
Rural	205 (44.8)	1.39 (1.09,1.77)	0.008	1.31 (1.01,1.70)	0.044
**Sex**					
Male	195 (33.9)	Ref		Ref	
Female	257 (46.5)	1.70 (1.33,2.16)	< 0.001	1.57 (1.21,2.07)	0.001
**Occupation**					
Student	40 (38.5)	Ref		Ref	
Unemployed	154 (45.7)	1.35 (0.86,2.11)	0.195	1.18 (0.61,2.29)	0.628
Business/service	103 (32.8)	0.78 (0.49,1.24)	0.292	0.98 (0.52,1.84)	0.945
Professional	101 (43.5)	1.23 (0.77,1.98)	0.384	1.34 (0.71,2.53)	0.364
Farmers and workers	54 (38.0)	0.98 (0.58,1.65)	0.945	1.18 (0.57,2.45)	0.654
**Education**					
Primary school and below	134 (43.4)	Ref		Ref	
Middle school	225 (38.7)	0.83 (0.62,1.09)	0.179	1.35 (0.92,1.98)	0.127
College and above	93 (38.9)	0.83 (0.59,1.17)	0.294	1.14 (0.70,1.86)	0.605
**Child under 5 years old**					
No	290 (41.0)	Ref			
Yes	162 (38.5)	0.90 (0.70,1.15)	0.411	0.97 (0.72,1.30)	0.829
**Income (yuan)**					
< 100,000	270 (40.5)	Ref		Ref	
100,000-199,999	134 (40.5)	1.00 (0.77,131)	0.999	1.08 (0.79,1.48)	0.644
≥ 200,000	47 (36.2)	0.83 (0.56,1.23)	0.357	0.99 (0.63,1.56)	0.968
**Smoking status**					
No	260 (43.9)	Ref		Ref	
Yes	192 (35.8)	**0.71 (0.56,0.90)**	**0.005**	0.81 (0.62,1.06)	0.126
**Chronic disease**					
No	285 (36.4)	Ref		Ref	
Yes	167 (48.3)	**1.63 (1.26,2.11)**	< 0.001	1.28 (0.93,1.77)	0.128
**Duration of cough (week)**					
≤ 2	372 (37.5)	Ref		Ref	
3–8	56 (68.3)	**3.59 (2.22,5.82)**	< 0.001	**2.81 (1.68,4.73)**	< 0.001
> 8	24 (43.6)	1.29 (0.75,2.23)	0.362	1.26 (0.70,2.26)	0.448
**Number of accompanying symptoms**					
0	96 (24.9)	Ref			
1	179 (42.1)	**2.19 (1.62,2.96)**	< 0.001	**2.19 (1.59,3.01)**	< 0.001
≥ 2	177 (55.5)	**3.75 (2.73,5.17)**	< 0.001	**3.87 (2.74,5.47)**	< 0.001

### Factors associated with choice of medical facility

Of the 452 participants who went for medical treatment, 120 (26.5%) chose hospitals at county level or above. The associations of socio-demographic characteristics and features of cough with choice of medical facility are presented in Table 3. Female and people live in rural areas were 0.33 (95% CI: 0.21, 0.54) and 0.57 (95% CI: 0.36, 0.92) times less likely to choose hospitals at county level or above respectively. Individuals with college degree or above were more likely to seek medical treatment in hospitals at or above the county level compared to those with education below primary school (OR = 3.29, 95% CI: 1.35, 8.02). Participants who coughed for 3–8 weeks and > 8 weeks were associated with a higher likelihood of choosing hospitals at or above the county level (OR = 2.35, 95% CI: 1.19, 4.61, OR = 3.13, 95% CI: 1.24, 7.90, respectively). Number of accompanying symptoms does not show significant relationship with choosing medical facility.

**Table 3:** Univariate and multivariate logistic regression analysis for choice of medical facility after coughing (*N* = 452)

Characteristics	Choice of Medical Facility		Crude OR	***P*** value	Adjusted OR	***P*** value
	Community health service center and below N (%)	hospital at county level and above N (%)				
**Age**						
15-24y	61 (70.9)	25 (29.1)	Ref		Ref	
25-64y	207 (77.5)	60 (22.5)	0.71 (0.41,1.22)	0.215	0.84 (0.39,1.81)	0.649
≥65y	64 (64.6)	35 (35.4)	1.33 (0.72,2.49)	0.363	2.72 (0.90,8.20)	0.075
**Residence**						
Urban	162 (65.6)	85 (34.4)	Ref		Ref	
Rural	170 (82.9)	35 (17.1)	**0.39 (0.25,0.62)**	**< 0.001**	**0.33 (0.21,0.54)**	**< 0.001**
**Sex**						
Male	135 (69.2)	60 (30.8)	Ref		Ref	
Female	197 (76.7)	60 (23.3)	0.69 (0.45,1.04)	0.077	**0.57 (0.36,0.92)**	**0.021**
**Occupation**						
Student	27 (67.5)	13 (32.5)	Ref		Ref	
Unemployed	112 (72.7)	42 (27.3)	0.78 (0.37,1.65)	0.514	0.73 (0.22,2.41)	0.61
Business/service personnel	76 (73.8)	27 (26.2)	0.74 (0.33,1.63)	0.453	0.65 (0.21,2.04)	0.457
Professional	78 (77.2)	23 (22.8)	0.61 (0.27,1.38)	0.235	0.43 (0.14,1.31)	0.137
Farmers and workers	39 (72.2)	15 (27.8)	0.80 (0.33,1.95)	0.621	0.74 (0.20,2.80)	0.659
**Education**						
Primary school and below	99 (73.9)	35 (26.1)	Ref		Ref	
Middle school	171 (76)	54 (24)	0.89 (0.55,1.46)	0.653	1.44 (0.69,3.01)	0.332
College and above	62 (66.7)	31 (33.3)	1.41 (0.79,2.52)	0.24	**3.29 (1.35,8.02)**	**0.009**
**Child under 5**						
No	206 (71)	84 (29)	Ref		Ref	
Yes	126 (77.8)	36 (22.2)	0.70 (0.45,1.10)	0.12	0.75 (0.42,1.34)	0.33
**Household income (yuan)**						
< 1 00,000	203 (75.2)	67 (24.8)	Ref		Ref	
100,000—199,999	100 (74.6)	34 (25.4)	1.03 (0.64,1.66)	0.903	0.98 (0.55,1.75)	0.937
≥ 200,000	28 (59.6)	19 (40.4)	2.06 (1.08,3.92)	0.028	1.74 (0.80,3.79)	0.161
**Smoking status**						
No	184 (70.8)	76 (29.2)	Ref		Ref	
Yes	148 (77.1)	44 (22.9)	0.72 (0.47,1.11)	0.134	0.73 (0.45,1.18)	0.193
**Chronic disease**						
No	210 (73.7)	75 (26.3)	Ref		Ref	
Yes	122 (73.1)	45 (26.9)	1.03 (0.67,1.59)	0.884	0.63 (0.35,1.14)	0.123
**Duration of cough (week)**						
≤ 2	284 (76.3)	88 (23.7)	Ref		Ref	
3–8	35 (62.5)	21 (37.5)	**1.94 (1.07,3.50)**	**0.029**	**2.35 (1.19,4.61)**	**0.014**
> 8	13 (54.2)	11 (45.8)	**2.73 (1.18,6.31)**	**0.019**	**3.13 (1.24,7.90)**	**0.016**
**Number of accompanying symptoms**						
0	69 (71.9)	27 (28.1)	Ref		Ref	
1	131 (73.2)	48 (26.8)	0.94 (0.54,1.63)	0.816	1.25 (0.66,2.36)	0.502
≥ 2	132 (74.6)	45 (25.4)	0.87 (0.50,1.52)	0.629	1.14 (0.59,2.18)	0.704

## Discussion

To the best of our knowledge, the present study is the first community-based study focused on the association of socio-demographic and cough characteristics with health seeking behavior in the Chinese population. We found that 19.3% (*n* = 5855) of the respondents reported a cough in the past month, 40% (*n* = 1129) of them had sought medical treatment. Individuals more than 65 years old, female, with cough for 3–8 weeks and with more accompanying symptoms were more likely to seek medical treatment. For those had sought medical treatment, 26.5% (*n* = 462) chose hospitals at county level or above. Male, people live in urban areas, individuals graduated from college and above and with cough for more than 2 weeks were more likely to choose hospitals at county level or above.

### Health-seeking decision

Contrary to some previous studies on health-seeking behavior [12, 14, 15], we found that some of the socio-demographic characteristics, such as education, occupation, family size and household income do not seem to be associated with the probability of seeking medical treatment after coughing. These may be related to population selection, and heterogeneity in different countries.

We also found that smokers were less likely to seek medical treatment in the univariate analysis, but the association became insignificantly after adjusting for factors such as age and duration of cough. Results of existing studies on the association between them after coughing were inconsistent [12, 14, 18], this may be due to different confounding factors adjusted in the model, and the characteristics of cough were not included in the multivariate analysis in most studies. Although the current research results were inconsistent, given the smoking rate in China [24], this is still a very worrying and important finding, suggesting that attention should be paid to the health seeking behavior of smokers after coughing, which may also contribute to the early diagnosis and treatment of lung cancer.

The present study found that individuals aged ≥65 years old and living in rural areas were more likely to seek medical treatment. Besides, women were more likely to seek medical treatment than men, which is in parallel with studies conducted in Zambia and Vietnam [15, 25]. This may be explained by the fact that women were more troubled by cough than men [26, 27]. What’s more, some studies focusing on sex differences in health-seeking behavior have pointed out that men were more likely to behave masculinity and a healthy state thus less likely to seek care [28].

The present study was the first to focus on the association between cough duration and health-seeking decision in China. A study in Finnish found that people with subacute and chronic coughs were more likely to seek medical attention [29]. However, in the present study, we found individuals with cough for 3–8 weeks were more likely to seek medical treatment, while those coughed for > 8 weeks were not. The possible explanation for this phenomenon could be that individuals who have been coughing for > 8 weeks were already used to coughing and regard it as a lifestyle habit rather than a disease. However, in addition to common causes of chronic cough, recent studies have found that pertussis has became a prevalent disease in some areas [7, 8], and B. pertussis infection should be considered as a significant pathogenic infection in adult patients presenting a cough of more than 3 weeks duration [30, 31]. Tuberculosis also should be evaluated among persons with unexplained productive cough lasting 2–3 weeks or more [6]. Thus there is no doubt that prolonged coughing without seeking medical treatment timely will bring an increased risk of transmission of such respiratory infectious disease. It is extremely desirable to undertake extensive educational campaigns about cough especially persistent one, so as to encourage individuals to visit medical facilities in time.

What’s more, the number of accompanying symptoms showed a clear increasing trend with health-seeking decision, as were observed in India and Tanzania [13, 20]. Coughing is generally considered to be a very common symptom rather than a disease, people do not usually seek medical care if a cough is not distressing or associated with any other symptoms that restrict one’s ability to function/work [32, 33]. Recent studies further demonstrated that an impaired cough-related quality of life is the most important determinant of the decision to visit a doctor due to cough [29, 34].

### Choice of medical facility

In the present study, the multivariate analysis consistently show that individuals living in rural areas and women were less likely to choose hospitals at or above the county level, while the most educated persons were more likely. Studies conducted in Vietnam [25] and China [23, 35] also found that women took more health-care actions than men, but chose less qualified providers (like self-medication, pharmacist, or private practitioner) and individuals living in rural areas had poor access to medical facility. But contrary to previous studies conducted in China [21, 22, 36], the household income was not significant associated with the choice of medical facility after adjusting for other confounders in the present study. This change may be due to a decline in health spending variance as the economy develops, with rising living standards and incomes.

We also found that individuals who coughed for more than 2 weeks were associated with a higher likelihood of choosing hospitals at or above the county level, while number of accompanying symptoms did not show any significant relationship with their choice. This reveals an interesting fact that people were more likely to seek medical treatment when the accompany symptoms of cough appear, but pay more attention to the duration of cough when choosing a hospital. The result echoes a call in a Japanese study [37], which suggested that individuals suffering from cough that does not resolve within a short period of time should be taken seriously regardless of the severity, as cough can be a sign of serious diseases such as lung cancer, pertussis and pulmonary tuberculosis [2, 38, 39]. Based on the fact, it is recommended to strengthen the training of relevant personnel in such facilities to improve their attention to persistent cough, as well as the level of surveillance and case detection of the above serious diseases.

### Limitations

Although the data in the present study was based on a community-based population and corrected for established and potential confounding factors (both socio-demographic and cough characteristics), these findings should also be interpreted in light of some limitations. First, it is important to acknowledge the limitations of the subjective perceptions and self-reports and therefore may lead to recall bias or be biased by potential under-reporting or over-reporting. Second, due to the limited sample size, some significant positive associations may not be demonstrated in the present study. Third, no respondents’ refusal was captured during the conduct of the survey, that may lead to a higher response rate than other survey. Finally, the study cannot exclude the effects of residual confounding by unmeasured risk factors, such as medical insurance and cause of cough. Therefore, larger sample size studies with more adjusted confounders are warranted to examine the further association and make a firm complement to the current study.

## Conclusion

The present study found that 40% of individuals sought medical treatment after cough and of these, about 1/3 chose hospitals at county level or above. Age, sex, living areas, education level as well as features of cough were associated with health seeking behavior. Targeted intervention measures should be formulated based on the above key factors to guide people to seek medical treatment scientifically and rationally.

## Supplementary Information

**Additional file 1.** Questionnaire.

## Data Availability

The datasets used and/or analyzed during the current study are available from the corresponding author on reasonable request.
