# Supplementary material for: Health seeking behavior and associated factors among individuals with cough in Yiwu, China: a population-based study
Source: BMC Public Health. 2021 Jun 16;21:1157. doi: 10.1186/s12889-021-11250-5 (PMC8207678; doi:10.1186/s12889-021-11250-5)
Supplement: Supplementary file 1 — Additional file 1. Questionnaire. [file 12889_2021_11250_MOESM1_ESM.docx]

**Questionnaire**

**Part 1 Demographic Characteristics**

- 1. **Date of birth:** yyyy mm dd

**1.2 Sex:** (1) male; (2) female

**1.3 Nation:** (1) Han; (2) others_________

**1.4 Occupation:**

(1) student; (2) housework and unemployment; (3) retired personnel; (4) Business/service personnel; (5) Food and catering industry employees; (6) Professionals (civil servants, teachers, medical staff, workers, etc.); (7) Practitioners in agriculture, forestry, animal husbandry and fishery; (8) others_________

**1.5 Education：**

(1) primary school and below; (2) junior high school; (3) senior high school; (4) technical secondary school; (5) junior college; (6) bachelor degree and above

**1.6 Including yourself, there are _____ people in your family (referring to all people who share the same kitchen or dining table), including _____ children under 5 years old.**

**1.7 Have you ever smoked?？** (1) yes; (2) Yes, but have quit smoking; (3) no

**1.8 Do you have any of the following chronic underlying diseases? (Multiple options)**

(1) yes; (2 ) no (Skip to the question 2.1)

□Cardiovascular and cerebrovascular diseases; □Chronic diseases of ear, nose and throat;

□hypertension; □diabetes; □Digestive system diseases; □Chronic respiratory diseases; □others

**1.8.1 If you have hypertension, are you currently taking antihypertensive drugs?**

(1) yes; (2 ) no

**Part 2 Cough characteristics and Health seeking behavior**

**2.1 Have you had a cough during the past month?**

(1) yes; (2 ) no (Skip to the question 2.2)

**If yes,**

**2.1.1 How long did your last cough last?**

(1) <1 week; (2) 1-2weeks; (3)3-4weeks; (4) 5-8 weeks; (5) >8 weeks

**2.1.2 Did you have the following symptoms during the last cough? (Multiple options)**

□ no; □ fever (Axillary temperature≥37.2℃); □ expectoration; □ runny nose; □ belching; □ irritability; □ vomit; □ headache; □ shortness of breath; □ earache; □ sore throat; □ dyspnea; □ abdominal pain; □ muscle or joint pain; □ chest pain; □ fatigue; □ lethargy; □ others____

**2.2 Did you go to a medical facility for treatment during your last cough (not limited to the past month)?**

(1) yes; (2 ) no

**2.2.1 If yes, which medical facility did you choose?**

□ municipal hospitals(Such as Yiwu Central Hospital, maternal and child health hospital,etc.)

□ 14 township hospitals or community health service centers

□ Clinics(Village clinic or private clinic)

□ others_________

**2.2.2 If no, why didn't you see a doctor****? (Multiple options)**

□ The symptoms were mild and did not require medical attention;

□ The hospital is too far from home and the transportation is inconvenient;

□ Drugs purchased in pharmacies; □ Don't trust the doctor; □ High medical expenses;

□ Too busy with work to have time; □ Medical expenses cannot be reimbursed;

□ Poor hospital facilities and environment; □ others_________
